# Fabrication of an SPR Sensor Surface with Antifouling Properties for Highly Sensitive Detection of 2,4,6-Trinitrotoluene Using Surface-Initiated Atom Transfer Polymerization

**DOI:** 10.3390/s130709294

**Published:** 2013-07-19

**Authors:** Rui Yatabe, Takeshi Onodera, Kiyoshi Toko

**Affiliations:** Graduate School of Information Science and Electrical Engineering, Kyushu University, 744 Motooka, Nishi-ku, Fukuoka 819-0395, Japan; E-Mails: onodera@ed.kyushu-u.ac.jp (T.O.); toko@ed.kyushu-u.ac.jp (K.T.)

**Keywords:** trinitrotoluene, surface plasmon resonance, immunosensor, surface initiated atom transfer polymerization, self-assembled monolayer, nonspecific adsorption

## Abstract

In this study, we modified a surface plasmon resonance immunosensor chip with a polymer using surface-initiated atom transfer polymerization (SI-ATRP) for the highly sensitive detection of 2,4,6-trinitrotoluene (TNT). To immobilize a TNT analogue on the polymer, mono-2-(methacryloyloxy)ethylsuccinate (MES), which has a carboxyl group, was used in this study. However, the anti-TNT antibody may adsorb non-specifically on the polymer surface by an electrostatic interaction because MES is negatively charged. Therefore, a mixed monomer with MES and diethylaminoethylmethacrylate (DEAEM), which has a tertiary amino group and is positively charged, was prepared to obtain electroneutrality for suppressing the nonspecific adsorption. The detection of TNT was performed by inhibition assay using the polymer surface. To ensure high sensitivity to TNT, the affinity between the surface and the antibody was optimized by controlling the density of the initiator for ATRP by mixing two types of self-assembled monolayer reagents. As a result, a limit of detection of 5.7 pg/mL (ppt) for TNT was achieved using the optimized surface.

## 1. Introduction

Recently, there has been increasing concern about terrorist attacks using bombs. Metal detectors, X-ray inspection apparatus, and sniffer dogs are mainly used to detect explosives. However, metal detectors and X-ray inspection apparatus cannot directly detect explosives. Sniffer dogs have some problems regarding their sensitivity errors because the dogs are living beings. To resolve these problems, it is necessary to detect explosive compounds using a sensor.

The detection of explosives has been attempted by various other methods. In one method, the detection was performed using a localized surface plasmon resonance (LSPR) sensor containing an imprinted polymer with gold nanoparticles [1]. The advantage of this method is its ultrahigh sensitivity to 2,4,6-trinitrotoluene (TNT). In addition, an electrochemical method employing SiO_2_ nanostructures was developed to detect nitroaromatic compounds [2]. This method has the advantage that the instrument used for detection is simple and suitable for constructing inexpensive and portable sensors. In addition to these methods, detection has been carried out using the quenching of fluorescence by nitroaromatic compounds [3,4]. The advantage of this method is that the explosive can be detected in air. Indeed, considerable effort has been made for the detection of TNT.

We previously realized an ultrahigh sensitivity TNT sensor using a surface plasmon resonance (SPR) sensor and an antigen-antibody interaction [5–8]. The SPR sensor, which is a high sensitivity refractive index sensor, can detect a change in the refractive index upon the adsorption of compounds on a gold surface. The antigen-antibody interaction is an immunoreaction with high selectivity to a target compound. The TNT sensor, which has high selectivity and sensitivity, operates via the combination of these principles.

It is advantageous to increase the number of binding sites of the TNT antibody for the detection of TNT at low concentrations. We realized the sensor surface by arranging the binding sites in three dimensions using a dendrimer or a polymer – poly-(vinylamine-co-*N*-vinylformamide) [9].

Recently, surface-initiated atom transfer polymerization (SI-ATRP) has been used as a method of surface modification [10]. This polymerization method has the following characteristics: first, straight-chain polymers are directly connected to a surface because the polymer chains grow from initiation points on the surface. Next, the polymerization reaction can restart because the growing points of the polymer chains are under a dormant condition even if the polymerization is stopped. It is easy to control the length of the polymer, and some types of polymers can connect to each other in series owing to this characteristic. To summarize, surfaces with various structures can be realized by SI-ATRP.

To realize a surface with antifouling properties, SI-ATRP has been used in the fabrication of zwitterionic polymer brushes. These brushes were used to measure a component in blood plasma without purification [11]. As another application of SI-ATRP, two types of polymer chains were grown in series from a surface to realize two different characteristics by living polymerization. The first polymer with a high packing density was prepared to resist the nonspecific adsorption of complex media. The second polymer with a low packing density was prepared to achieve a high protein-binding capacity [12]. Another application is the amplification of SPR signals. Signal amplification was performed using a polymer grown from an antibody adsorbed on a surface [13]. In conclusion, the SI-ATRP technique has been employed for the fabrication of various types of surfaces.

In this study, we attempted to fabricate an electrically neutral polymer surface using SI-ATRP as the method to arrange the binding sites in three dimensions. A mixed monomer consisting of two types of monomer was used for the fabrication of the polymer surface with resistance to nonspecific adsorption. One of them was mono-2-(methacryloyloxy)ethylsuccinate (MES), which has a carboxyl group on the side chain. A TNT analogue was immobilized to the carboxyl group as a binding site of the anti-TNT antibody. The other monomer was diethylaminoethylmethacrylate (DEAEM), which has a tertiary amino group on the side chain. The polymer surface with low nonspecific adsorption was fabricated using the mixed monomer. The limit of detection (LOD) for TNT was evaluated on the SPR sensor modified with the polymer surfaces of various densities.

## 2. Experimental Section

### 2.1. Materials

Bis[2-(2′-bromoisobutyryloxy)undecyl]disulfide (DTBU) was purchased from Sigma Aldrich (St. Louis, MO, USA) to form a self-assembled monolayer (SAM) as an initiator for ATRP. 11-mercaptoundecanol triethyleneglycol ether (Hydroxyl EG3 undecanethiol; HEg_3_UT) was obtained from Dojindo Laboratories (Kumamoto, Japan) to form a SAM that did not act as an initiator for ATRP. *N*,*N*,*N*′,*N*”*N*”-pentamethyldiethylenetriamine (PMDETA, Tokyo Chemical Industry Co., Tokyo, Japan), copper bromide (CuBr_2_, Nacalai Tesque, Inc., Kyoto, Japan), and ascorbic acid (Wako Pure Chemical Industries, Osaka, Japan) were used in the ATRP reaction. MES and DEAEM were prepared as monomer from Sigma Aldrich. *N*-hydroxysuccinimide (NHS) and 1-ethyl-3-(3′-dimethylaminopropyl) carbodiimide hydrochloride (EDC) were purchased as an amine coupling kit from GE Healthcare Bio-Sciences (Uppsala, Sweden). 2,4-dinitrophenyl-*e*-aminocaproyl-NHNH2 (DNP-Hdrz) was obtained as a TNT analogue from Biosearch Technologies, Inc. (Novato, CA, USA). Mouse anti-TNT monoclonal antibody (anti-TNT antibody) was purchased from Strategic Biosolutions (Newark, DE, USA). A TNT aqueous solution with a concentration of 21.8 ppm was obtained from Chugoku Kayaku (Hiroshima, Japan). Bovine serum albumin (BSA, Wako Pure Chemical Industry) and lysozyme (Sigma Aldrich) were used to evaluate nonspecific adsorption. All other chemicals were purchased either from Tokyo Chemical Industry,Wako Pure Chemical Industries, Inc. or Kanto Chemical, Co., Tokyo, Japan. All aqueous solutions were prepared from Milli-Q water obtained from a Milli-Q system (Millipore, Billerica, MA, USA).

### 2.2. Fabrication of Sensor Chip Surface

**Scheme 1.**
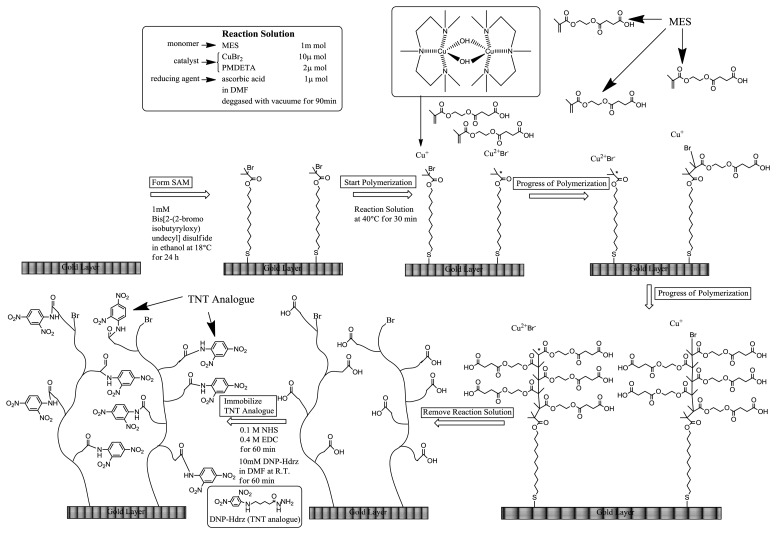
Fabrication procedure of polymer-based sensor surface by SI-(AGET)ATRP.

The SIA Kit Au (GE Healthcare Bioscience), which contains sensor chips with an unmodified gold layer of ca. 50 nm thickness, was used for the immobilization of various reagents on the surface. Scheme 1 shows the fabrication procedure of a sensor surface using SI-ATRP. This scheme shows the case in which the monomers were not mixed to simplify the explanation. First, the sensor chip was cleaned in a mixed solution of Milli-Q water, ammonia solution, and hydrogen peroxide with a 5:1:1 volume ratio at 90 °C for 20 min. After that, the sensor chip was immersed in 1 mM DTBU (in ethanol) for 24 h at 18 °C to form a SAM with an initiator for ATRP. Next, the sensor chip was immersed in a reaction solution at 40 °C to induce activator-generated electron transfer atom transfer radical polymerization (AGET-ATRP). The reaction solution contained a monomer solution, a catalyst, and a reducing agent in a molar ratio of 1000:2:1. The monomer solution (MES in Scheme 1) was diluted with same volume of *N*,*N*-dimethylformamide (DMF). The catalyst was a mixed solution of CuBr_2_ and PMDETA with a molar ratio of 1:1 and a concentration of 100 mM in DMF. The reducing agent was a 100 mM ascorbic acid solution in DMF. These solutions were degassed in vacuum for 1.5 h, and they were mixed immediately before the polymerization. After the polymerization, the reaction solution was removed using DMF and water. The thickness of polymer layer in air was measured by Spectroscopic Ellipsometer SpecEl-2000-VIS (Mikropack GmbH, Ostfildern, Germany). Then, the sensor chip was immersed in a mixed solution of 0.4 M EDC (in water) and 0.1 M NHS (in DMF) with a 1:1 volume ratio for 1 h to convert the carboxyl groups of the polymer to NHS esters. Next, the chip was immersed in 10 mM DNP-Hdrz for 1 h to combine the amino group of the DNP-Hdrz and the carboxyl group of the polymer. Then, the chip was immersed in 0.5 wt% sodium dodecyl sulfate for cleaning. Finally, it was rinsed in Milli-Q water to complete the fabrication of the sensor chip with binding sites of an anti-TNT antibody on the polymer.

### 2.3. Instrument and Conditions for SPR

Biacore J (GE Healthcare Bio-sciences) was used for the SPR measurement. HBS-T (10 mM 2-[4[-(2-hydroxyethyl)-1-piperazinyl]-ethane sulfonic acid, 150 mM NaCl, 0.005% Tween 20: pH 7.4) was used as a running buffer solution. The measurements were conducted at a constant temperature of 25 °C in nitrogen. The flow rate of the solution was 30 μL/min. When the anti-TNT antibody solution was allowed to flow over the DNP-Hdrz immobilized on the chip surface, the antibody is bound to the DNP-Hdrz. The SPR sensor can measure a change in the refractive index. The SPR response, which is defined in resonance units (RU), increased with increasing amount of antibody bound on the surface. A resonance angle shift of 0.1 is defined as 1,000 RU and is equivalent to a change in mass of 1 ng/mm^2^ on the surface [14]. In most of the experiments, the dissociation of surface-bound antibodies for regeneration was completed when 5 M guanidine hydrochloride was allowed to flow over the surface. The association rate constant (K_a_) and dissociation rate constant (K_d_) were calculated from the sensor response curve by BIAevaluation software (version 3.2; GE Healthcare Bio-sciences). The analysis model was 1:1 (Langmuir) binding. To induce association, the antibody with 200 ppb concentration was allowed to flow for 6 min. The dissociation was conducted for 6 min.

### 2.4. TNT Detection Using the Inhibition Assay

TNT detection was carried out by inhibition assay [7,8,15]. An antibody solution of known concentration was mixed with a TNT solution. An anti-TNT antibody interacts with TNT by specific binding. The mixed solution was immediately allowed to flow on the surface. Then, the anti-TNT antibodies that did not react with TNT were bound to the DNP-Hdrz on the chip. The sensor response was obtained by the binding of anti-TNT antibodies to the DNP-Hdrz on the chip. The TNT concentration of a sample solution was determined from the ratio between the response of an antibody solution without TNT and that of the solution with TNT. The ratio is called “Bound percentage”. The concentration of the antibody used was 100 ppb (ng/mL). The solutions were allowed to flow for 6 min.

## 3. Results and Discussion

### 3.1. Control of Nonspecific Adsorption on Poly-MES-Co-Poly-DEAEM Based Surface

Our TNT sensor indirectly detects TNT using specific binding with a TNT analogue and an antibody that does not bind to TNT. Therefore, it is difficult to detect TNT when nonspecific adsorption, which is not the antigen-antibody interaction, occurs because the SPR sensor cannot distinguish between specific and nonspecific adsorption. Thus, it is necessary to control the nonspecific adsorption.

There are two mechanisms of nonspecific adsorption. First, it is a hydrophobic interaction. When a surface has hydrophobic parts, the antibody adsorbs on the surface via each hydrophobic parts because the measurement is performed in an aqueous phase. Second, it is an electrostatic interaction. The antibody is positively or negatively charged in the aqueous phase. Therefore, when the surface is oppositely charged, the antibody is adsorbed on the surface. Therefore, the surface must be hydrophilic and electrically neutral.

We fabricated a polymer surface using two types of monomer to obtain such properties. MES is a negatively charged monomer with a carboxyl group on the side chain. DEAEM is a positively charged monomer with a tertiary amino group on the side chain. The DNP-Hdrz was attached with the carboxyl group of MES. The negative charge of unreacted carboxyl group in MES was neutralized by the tertiary amino group of DEAEM. We attempted to control the nonspecific adsorption by changing the mixing ratio of their monomers.

The surface was fabricated with the method described in Section 2.2. The polymerization time of a mixed monomer was adjusted to coordinate the thickness of polymer layer. The thickness was about 20 nm in air. After the polymerization, the DNP-Hdrz was immobilized to polymer chain. The fabrication of surface was completed with the method of Section 2.2.

**Figure 1.**
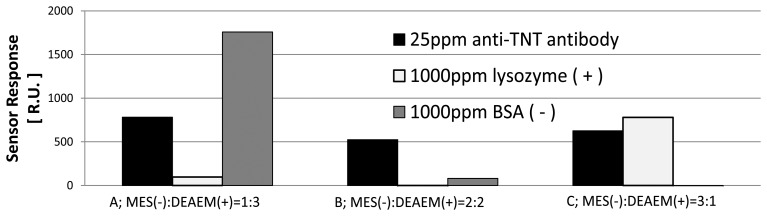
Adsorption of lysozyme, BSA and anti-TNT antibody.

The amount of adsorption on the surface was measured by SPR when 25 ppm (μg/mL) anti-TNT antibody, 1,000 ppm lysozyme and 1,000 ppm BSA were allowed to flow over the surface for 2 min. These proteins were dissolved in HBS-T buffer solution. If the carboxyl group in polymer still were activated by NHS ester, these proteins would be chemically immobilized after injection of these solutions to the surface. However, the chemical immobilization was not observed because the adsorption of these proteins was able to dissociate by flowing 50 mM NaOH solution for 2 min. Anti-TNT antibody adsorbs on the sensor surface by specific or nonspecific binding. Lysozyme is positively charged and adsorbs on the surface by nonspecific adsorption when the surface is negatively charged. BSA is negatively charged and adsorbs by nonspecific adsorption when the surface is positively charged. Figure 1 shows the SPR sensor response when the proteins were allowed to flow over the surfaces fabricated using the mixed monomer. The ratio of MES:DEAEM was prepared with 1:3, 2:2, and 3:1. When the amount of DEAEM was higher than the amount of MES, more BSA was adsorbed than the other proteins because the surface was positively charged. Conversely, when the amount of MES was higher than the amount of DEAEM, more lysozyme was adsorbed than the other proteins because the surface was negatively charged. On the other hand, when the amounts of MES and DEAEM were the same, there was little adsorption on the surface. A surface with low nonspecific adsorption was thus realized by changing the ratio of the mixed monomer.

The ratio of the mixed monomer was changed by small increments. The results are shown in Table 1. In MES:DEAEM ratios of 23:17, 24:16, or 25:15, the amount of the antibody adsorbed was larger and the nonspecific adsorption was lower than other ratios. We selected an MES:DEAEM ratio of 24:16 on the basis of the fabrication process to ensure stable properties of the sensor surface. The nonspecific adsorption was lowest under this condition for which the amount of MES was higher than the amount of DEAEM. The amount of MES must be higher to realize an electrically neutral surface because some of the carboxyl groups of MES were consumed in the reaction with the amino group of the TNT analogue.

### 3.2. Binding Capacity of Anti-TNT Antibody for Different Densities of Initiated SAM

The polymer chain starts to grow from an initiator SAM on our sensor surface by the ATRP reaction. On the other hand, it is known that the SAM is packed on the surface with a high density [16]. Thus, it is possible that the polymer on the surface is formed with a high density because the initiator for ATRP has a high density on the surface. There is a possibility that the antibody cannot move into the interior of such a polymer layer if steric hindrance exists, even if the antibody solution is allowed to flow over the surface. In this case, the antibody only binds to the sites on the outermost surface. The sensitivity of the SPR signal decreases with increasing distance from the gold surface. It is difficult to detect the binding of the antibody far from the gold surface by SPR. In conclusion, the binding capacities of the TNT antibody were evaluated on sensor surfaces modified with various densities of the polymer layer.

The density of the polymer layer was controlled by a mixed SAM. The mixed SAM was formed by mixing two types of SAM reagent solution with various molar ratios. One of them, DTBU, is an initiator for ATRP. The other was HEg_3_UT, which is not an initiator for ATRP. The sensor surfaces were fabricated using various ratios of mixed SAM by the method described in Section 2.2. The polymerization times were 300 s and 600 s to evaluate the effect of polymer chain length in parallel. The thicknesses of the polymer layer fabricated with the ratio of DTBU:HEg_3_UT = 1:0 were 44 nm and 118 nm in air, respectively. The amount of antibody bound on the surfaces was measured by the SPR sensor when the antibody with 25 ppm concentration was allowed to flow for 2 min. Table 2 shows the results. The amount of antibody bound on the surface increased with increasing ratio of HEg_3_UT to DTBU because the antibody can penetrate into the polymer layer owing to its low density. In conclusion, the binding capacity of the antibody can be controlled by changing the ratio of the mixed SAM.

### 3.3. Highly Sensitive Detection of TNT

**Figure 2.**
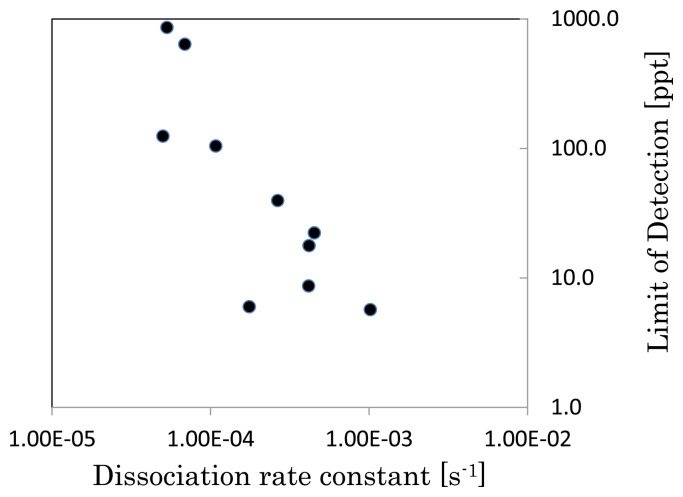
Relation of LOD and dissociation rate constant.

The detection of TNT was performed by inhibition assay. The LOD, K_a_, and K_d_ were determined for the previously described surfaces. The LOD was calculated by considering three standard deviations at the lowest concentration of TNT. These results were summarized in Table 3. The ratio of DTBU to HEg_3_UT increases K_d_. When the polymer density is low like ‘DTBU: HEg_3_UT = 1:100,000’, the antibody can penetrate deeply inside the polymer. The deep penetrated antibody can be bound again by TNT analogue even if the antibody may dissociate from TNT analogue once because the antibody is bound by many sites until the antibody get out from the polymer layer. As a result, K_d_ between the polymer surface and the antibody became low because the antibody in the polymer layer can hardly get out from the layer. Conversely, when the polymer density is high like ‘DTBU: HEg_3_UT= 1:10’, the antibody can exist near the polymer-outside interface because the antibody hardly penetrates into the inside of polymer layer due to steric hindrance. As a result, K_d_ became high because the antibody in the polymer layer easily get out from the layer. Figure 2 shows the relation between K_d_ and the LOD in Table 3. A lower LOD was realized when K_d_ was high. It indicates that highly sensitive detection is performed when the antibody dissociates from the surface easily.

**Figure 3.**
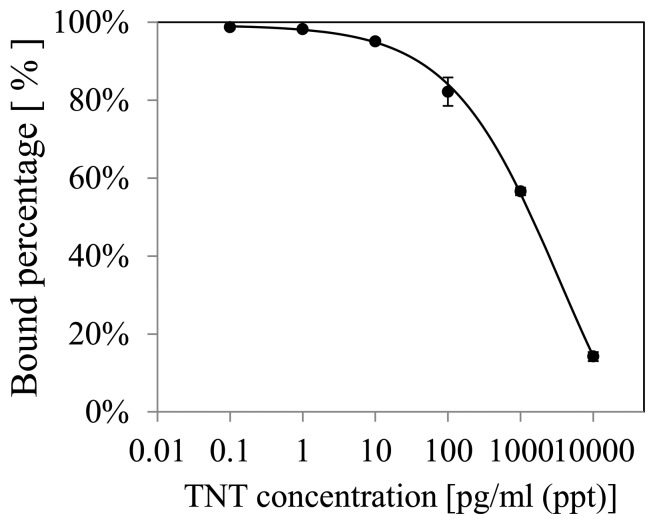
Response characteristic to TNT: Calibration curves obtained by inhibition assay using 100 ng/mL (100 ppb) anti-TNT antibody. The error bar shows the SD of the data.

From these results, the lowest LOD was 5.7 ppt (pg/mL) when the sensor surface was fabricated using the mixed SAM and mixed monomer. The ratio of the mixed SAM was DTBU:HEg_3_UT = 1:10. The ratio of the mixed monomer was MES:DEAEM = 24:16. To adjust the reaction rate, the mixed monomer was diluted with same volume of DMF. The polymerization time was 300 s. Figure 3 shows the obtained calibration curve. In an inhibition assay, it is difficult to achieve high sensitivity when the affinity between the surface and the antibody is too low or too high. When the affinity is too low, the SPR sensor response becomes low because few antibodies are adsorbed to the surface. When the affinity is too high, the SPR sensor response hardly depends on the concentration of TNT because the binding between the antibody and TNT analogue on the surface is rarely inhibited by TNT molecules. In other words, SPR measurement is difficult with too low affinity between the antibody and the surface, and inhibition assay or indirect assay is difficult with too high affinity. This is consistent with previous studies of our group [17]. In conclusion, highly sensitive detection was carried out by optimization of the affinity by changing the ratio of the mixed SAM.

## 4. Conclusions

We have fabricated an SPR sensor surface modified with a polymer by SI-ATRP for TNT detection. Nonspecific adsorption on the surface was controlled using a positively charged monomer and a negatively charged monomer because an electrically neutral and hydrophilic surface was realized. The affinity between the polymer surface and the anti-TNT antibody was optimized by changing the ratio of the mixed SAM. The lowest LOD for TNT was 5.7 ppt using the polymer surface. We plan to fabricate a new sensor surface using the characteristic of SI-ATRP that makes it possible to catenate heterogeneous polymers. We expect that a surface can be obtained that not only responds to the adsorption of antibody, but also to changes the polymer structure.

## Figures and Tables

**Table 1.** Optimization ratio of MES and DEAEM.

**Molar Ratio**		**SPR Sensor Response [R.U.]**		
	
MES	DEAEM	25 ppm Anti-TNT Antibody	1,000 ppm Lysozyme	1,000 ppm BSA
18	22	809.8	−49.3	18.4
20	20	854.9	−48.7	−13.7
22	18	677.5	−34.4	−17.1
23	17	1,150.2	6.9	−17.3
24	16	1,375.7	−58.4	−29.8
25	15	1,654.1	23.5	−50.7
27	13	1,221.9	78.4	−98.3

**Table 2.** Dilution ratios dependence of initiated SAM and their polymerization times.

**Binding Amount of Anti-TNT Antibody [R.U.]**		

**DTBU:HEg_3_UT**= 1: x	**Polymerization Time [s]**	

	300	600
10	554.1	819.3
100	1,052.7	848.0
1000	663.1	971.5
10,000	1,742.8	2,004.0
100,000	1,762.6	2,038.2

1 R.U. is the adsorption of protein with 1 pg/mm^2^ on the sensor surface.

**Table 3.** Kinetics parameters and the limit of detection (LOD).

**DTBU: HEg_3_UT = 1: x**	**Polymerization Time [s]**	**Association Rate Constant K_a_ [Ms^−1^]**	**Dissociation Rate Constant K_d_ [s^−1^]**	**Association Constant K_A_ [M]**	**LOD [ppt]**
10	300	1.76E + 06	1.02E − 03	1.73E + 09	5.7
10	600	4.32E + 05	4.19E − 04	1.03E + 09	17.7
100	300	8.02E + 05	1.76E − 04	4.56E + 09	6.0
100	600	9.21E + 03	4.16E − 04	2.21E + 07	8.7
1,000	300	1.24E + 06	4.53E − 04	2.74E + 09	22.2
1,000	600	1.07E + 04	2.66E − 04	4.02E + 07	39.5
10,000	300	1.53E + 05	5.02E − 05	3.05E + 09	124.4
1,000	600	2.44E + 05	6.90E − 05	3.54E + 09	638.3
100,000	300	2.81E + 05	1.08E − 04	2.60E + 09	104.2
100,000	600	4.17E + 05	5.32E − 05	7.84E + 09	859.1
